# Significant Effect of a Pre-Exercise High-Fat Meal after a 3-Day High-Carbohydrate Diet on Endurance Performance

**DOI:** 10.3390/nu4070625

**Published:** 2012-06-27

**Authors:** Ikuma Murakami, Takayuki Sakuragi, Hiroshi Uemura, Hajime Maeda, Munehiro Shindo, Hiroaki Tanaka

**Affiliations:** 1 Faculty of Sports and Health Science, Fukuoka University, 8-19-1 Nanakuma Jonan-ku, Fukuoka City, Fukuoka 814-0180, Japan; Email: murakami_ikuma@kurume-u.ac.jp (I.M.); takayuki_sakuragi_56@yahoo.co.jp (T.S.); hroshi_uemura1982@yahoo.co.jp (H.U.); mendy_h58@yahoo.co.jp (H.M.); m-shindo69@jcom.home.ne.jp (M.S.); 2 Institute for Physical Activity, Fukuoka University, 8-19-1 Nanakuma Jonan-ku, Fukuoka City, Fukuoka 814-0180, Japan

**Keywords:** marathon, glycogen, fat oxidation, carbohydrate oxidation, metabolism

## Abstract

We investigated the effect of macronutrient composition of pre-exercise meals on endurance performance. Subjects consumed a high-carbohydrate diet at each meal for 3 days, followed by a high-fat meal (HFM; 1007 ± 21 kcal, 30% CHO, 55% F and 15% P) or high-carbohydrate meal (HCM; 1007 ± 21 kcal, 71% CHO, 20% F and 9% P) 4 h before exercise. Furthermore, just prior to the test, subjects in the HFM group ingested either maltodextrin jelly (M) or a placebo jelly (P), while subjects in the HCM ingested a placebo jelly. Endurance performance was measured as running time until exhaustion at a speed between lactate threshold and the onset of blood lactate accumulation. All subjects participated in each trial, randomly assigned at weekly intervals. We observed that the time until exhaustion was significantly longer in the HFM + M (*p* < 0.05) than in HFM + P and HCM + P conditions. Furthermore, the total amount of fat oxidation during exercise was significantly higher in HFM + M and HFM + P than in HCM + P (*p* < 0.05). These results suggest that ingestion of a HFM prior to exercise is more favorable for endurance performance than HCM. In addition, HFM and maltodextrin ingestion following 3 days of carbohydrate loading enhances endurance running performance.

## 1. Introduction

Glycogen is an important source of energy for endurance exercise, such as marathons [1,2]. It is well-documented that prolonged exercise is associated with the depletion of the muscle glycogen in the working muscle. Thus, an increased glycogen concentration before exercise improves endurance performance [1]. The ingestion of a high-carbohydrate diet approximately 3 h before exercise can increase glycogen concentrations in muscle [3] and liver [4], thereby contributing to normal blood glucose concentrations during exercise. Endurance athletes were recommended to employ the carbohydrate-loading method [5,6] and ingest a high-carbohydrate meal (HCM) on the day of a race to enhance carbohydrate storage [7]. However, a study of Saltin *et al.* indicates that glycogen storage levels can peak after glycogen loading so that muscle glycogen may not further increase, even if large amounts of carbohydrates are ingested on race day [5]. Moreover, if there is an intake of high levels of carbohydrates before exercise, but after glycogen loading, then a rise in blood glucose might occur, resulting in an elevation of insulin that may persist. This, in turn, will inhibit free fatty acid (FFA) mobilization and may lead to the rapid depletion of glycogen during exercise, thereby negatively affecting performance [8].

Conversely, the intake of a high-fat meal (HFM) before exercise increases blood FFA levels when compared with those levels derived from ingestion of a HCM [9]. Increased blood FFA concentration contributes to an increase in lipid metabolism, thus resulting in the advantageous effect that muscle glycogen levels are conserved during endurance exercise [8,10,11,12]. As a result, a pre-exercise HFM may help to conserve carbohydrates, and consequently improve the endurance performance. However, this result was not demonstrated in human studies, with studies failing to show a difference in exercise performance between the consumption of a HCM and HFM [9,13]. These unexpected results may be due to the relationship between HFM diet and super-compensation of glycogen in the muscle; a hypothesis that has been neglected in the previous studies.

The intake of carbohydrate drinks just before and during prolonged exercise enhances performance [14,15,16,17,18,19]. We hypothesize that if glycogen loading [5,6] is carried out and the amount of glycogen stored reaches its maximum, the intake of a HFM on race day, which includes carbohydrates to replace hepatic glycogen that has been used during sleep, may help to improve performance as compared with the intake of a HCM. Moreover, ingestion of carbohydrates just before starting exercise would be expected to have a conservation effect on muscle glycogen, thereby further enhancing performance.

The purpose of this study was to investigate the effects of a HFM and a HCM 4 h prior to exercise after ingesting a high-carbohydrate diet for 3 days, based on previous studies [9,13], and to demonstrate the effects on endurance performance from ingesting carbohydrates immediately before exercise in subjects that have ingested a pre-exercise HFM.

## 2. Experimental Methods

### 2.1. Subjects

This study evaluated the impact of high fat or high carbohydrate diet prior to an endurance running test. Eight male collegiate long-distance athletes, who engaged in physical training almost every day, were recruited for the investigation. This study was approved by the Fukuoka University Ethical Committee. Written informed consent was obtained from all subjects. 

### 2.2. Preliminary Exercise Tests

The anthropometric characteristics of each subject were measured at least 1 week before the main trials. The body fat mass and ratio were measured by hydrostatic weighing, based on the hydrostatic density, with corrections made for the residual lung volume. The speed corresponding to the lactate threshold (LT) [20], the onset of blood lactate accumulation (OBLA), which is the theoretical anaerobic threshold [21], and the maximum oxygen intake (VO_2max_), were determined using an intermittent, multistep, increasing load exercise test on a treadmill (ELG-2, Woodway, WI) with 4-min and 2-min rests per single load. An initial load of 220 m/min was increased by 20 m/min per single load. A blood sample was obtained from the ear lobe at each stage to measure the lactic acid (LA) concentration (Biosen 5040, EFK, Germany). When the LA concentration exceeded 4 mmol/L, the load was increased at a continuous rate of 10 m/min until the subject reached exhaustion. The VO_2__max_ was measured from the mixed expired gas collected in neoprene bags. The volume of the expired gas was quantified with a twin-drum respirometer (Fukuda Irika CR-20, Tokyo, Japan). The O_2_ and CO_2_ fractions were analyzed by mass spectrometry (ARCO-1000, ARCO System Inc., Chiba, Japan). The O_2_ and CO_2_ fractions were calibrated using standardized gas (Approx. 16% O_2_ and 4% CO_2_). The characteristics of the subjects are shown in Table 1.

**Table 1:** Characteristics of the subjects.

**Age (y** **ear)**	22.2 ± 0.2
**Height (cm)**	169.2 ± 1.6
**Body mass (kg)**	55.9 ± 1.5
**Body fat (%)**	6.7 ± 0.7
**VO_2max_ (mL/kg/min)**	61.3 ± 2.2
**LT speed (m/min)**	254 ± 3.4

Abbreviations: VO_2max_, maximal oxygen uptake; LT speed, speed at the lactate threshold. Values shown as mean ± S.E.

### 2.3. Nutritional Status

Each subject ingested a HCM (2562 ± 19 kcal/day in total calories: 71% carbohydrates, 19% fat, and 10% protein) at all three meals for 3 days before the main trials. For the first 2 days during this time frame, training was limited to a low intensity and a low amount (far below the LT and within 30 min). In order to reach the near-maximum muscle glycogen storage amounts, the subjects were instructed to ingest a HCM and refrain from training on the day before measurement.

### 2.4. Main Trials

**Figure 1:**
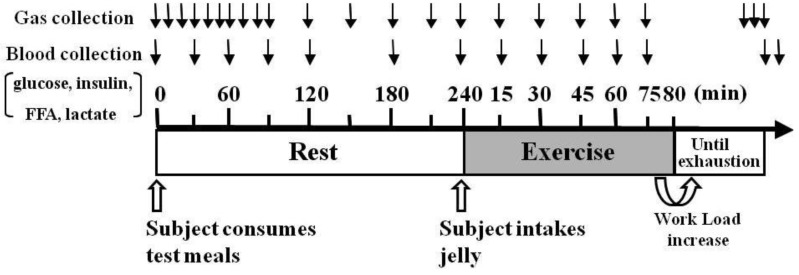
Experimental protocol. Protocol timeline illustrates gas collection, blood collection, exercise intensity periods and timing of ingesting high-fat or high-carbohydrate meal (test meals) 4 h before exercise, and either maltodextrin jelly or placebo jelly 3 min before exercise. Abbreviations: FFA, free fatty acid.

The protocol timeline is illustrated in Figure 1. The testing protocols were preceded by a 10-h overnight fast, and the subjects were randomly served either HFM (1007 ± 21 kcal/meal in total calories; 30% carbohydrates, 55% fat, and 15% protein) or HCM (1007 ± 21 kcal/meal in total calories; 70% carbohydrates, 21% fat, and 9% protein) 4 h before starting exercise. Three min before the trial, the subjects of the HFM group ingested either maltodextrin jelly (M: 410 ± 8 kcal), which has a higher rate of absorption than glucose, or placebo jelly (P: 0 kcal); subjects in the HCM group ingested placebo jelly. Subject allocation to M or P groups was completed in a double-blind method. All subjects participated in three trials (HFM + M, HFM + P, and HCM + P), with at least 1 week between each trial. The order for group participation for each subject was randomly assigned at the start of the study. The 80-min fixed load test was conducted using a treadmill at the LT speed (71.8 ± 1.7% VO_2max_), which conforms to each individual’s marathon race pace [22]. Next, each subject performed continuous endurance running until exhaustion at a speed between the LT and OBLA (80.0 ± 1.6% VO_2max_). The speed was employed to induce exhaustion by glycogen depletion, but not LA over accumulation. The heart rate (HR) was recorded by a heart rate monitor (Polar: Canon-trading Co., Inc., Tokyo, Japan), and included the HR at rest and the HR for 30 s before the conclusion of each exercise stage. Borg’s scale [23] was used to determine the rating of perceived exertion (RPE) immediately after the conclusion of each exercise stage. Subjects were permitted to voluntarily drink water during HFM and HCM ingestion and throughout the exercise period. The temperature and humidity in the laboratory were controlled to 20 °C and 56%, respectively. 

### 2.5. Measurement of Gas Exchange

Expired gas was collected in neoprene bags for 5 min after the subjects arrived at the laboratory and rested for 30 min, and for 5 min each at 10-min intervals up to 90 min after the subjects finished their meals and at 30-min intervals for an additional 240 min. In addition, during exercise, expired gas was collected for 1 min for every 15 min up to 75 min and for 2 min as the subject neared exhaustion. The collected expired gas volume was measured by a two-barrel drum-type respirometer and O_2_ and CO_2_ concentration in expired gas was measured by mass spectrometry. Carbohydrate and fat oxidation were calculated from VO_2_ based on the Lusk formula [24], as follows: (kcal/min) = VO_2_ (mL/min) × (3.81 + 1.23 × R)/1000.

### 2.6. Blood Samples Analysis

During the exercise, blood samples were taken every 15 min from the ear lobe of each subject and used to measure LA. Otherwise, blood samples were collected from the antecubital vein to measure blood glucose, insulin, FFA, and LA at fasting, and 30, 60, 90, 120, 180 and 240 min after meal ingestion. The LA value was analyzed using a lactate analysis device. Plasma and serum specimens were obtained following centrifugation at 3000 rpm for 10 min at 4 °C and stored at −80 °C until analysis. The plasma glucose concentrations were determined by UV-methods with hexokinase using Bio Majesty (JCA-BM 8000 series, JEOL, Tokyo, Japan). The insulin concentrations in the serum were determined with Enzyme Immunoassay using Beads Chemiluminescent System (BCS620, SRL Inc., Tokyo, Japan). The FFA concentrations were determined using an Enzymatic Method on Bio Majesty (JCA-BM2250, JEOL, Tokyo, Japan).

### 2.7. Statistical Analysis

Data from the three trials were analyzed using a two-way (meal and time) ANOVA with repeated measurements. One-way ANOVA was used to compare the performance times and to compare energy expenditure amongst the three groups. When significant differences were revealed using the ANOVA, a Tukey *post hoc* test was performed. All statistical analyses were conducted using Stat View software (version 5.0.1, SAS Institute, NC). Statistical significance was defined as being represented by a *p* value less than 0.05.

## 3. Results

The average time until exhaustion was significantly extended in subjects who ingested HFM + M as compared with those who ingested HFM + P or HCM + P (*p* < 0.05) (Table 2). The time until exhaustion for subjects who ingested HFM + P showed no significant differences when compared with subjects who ingested HCM + P, but the performance time of seven out of eight subjects was extended.

**Table 2:** Runningtime to exhaustion for three trials. HFM: high-fat meal; HCM: high-carbohydrate meal; M: maltodextrin; and P: placebo jelly.

	HFM + M	HFM + P	HCM + P
A	87	75	85
B	110	91	85
C	90	90	84
D	92	91	88
E	101	96	94
F	107	96	93
G	99	95	94
H	115	105	97
Mean ± SE	100 ± 3.4 *^,†^	92 ± 2.8	90 ± 1.7

Running time shown as minutes. * compared with HFM + P (*p* < 0.05). ^†^ compared with HCM + P (*p* < 0.05).

**Figure 2:**
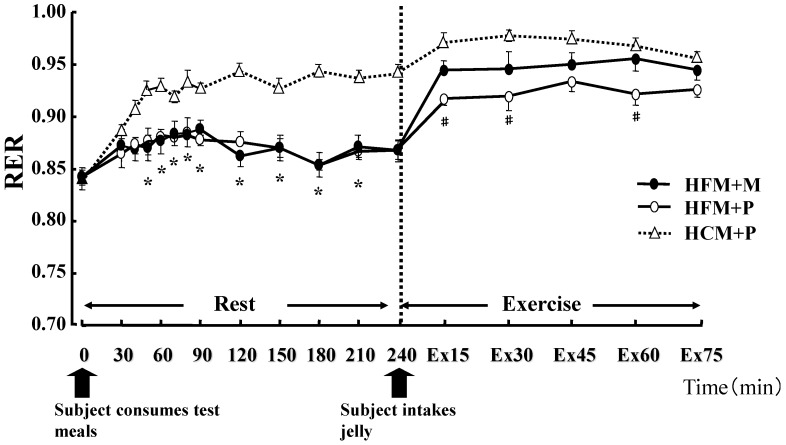
Changes in the Respiratory Exchange Ratio (RER) at rest and during exercise. * indicates when high-fat meal with maltodextrin (HFM+M) and high-fat meal with placebo (HFM+P) values were significantly lower than high-carbohydrate meal and placebo (HCM+P) values (*p* < 0.05). ^# ^indicates when HFM+P was significantly lower than HCM+P (*p* < 0.05). Data are shown asmean ± S.E.

**Figure 3:**
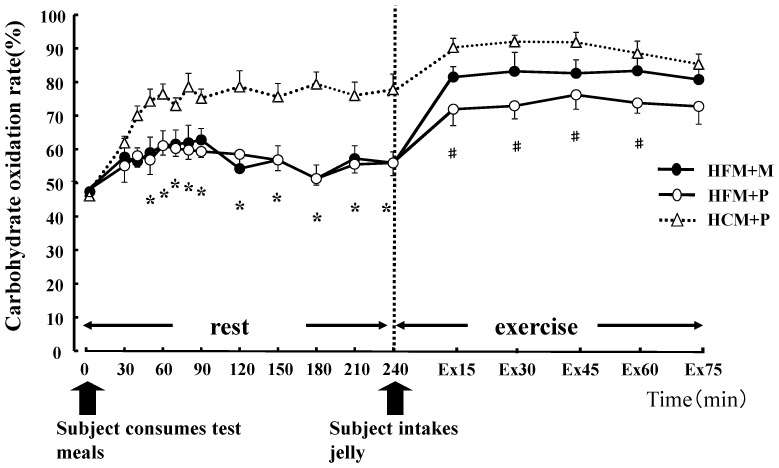
Carbohydrate oxidation rates (%) for 240 min at rest and for 75 min during exercise. * indicates when high-fat meal with maltodextrin (HFM+M) and high-fat meal with placebo (HFM+P) values were significantly lower than high-carbohydrate meal and placebo (HCM+P) values (*p* < 0.05). ^#^ indicates when HFM + P was significantly lower than HCM+P (*p* < 0.05). Data are shown asmean ± S.E.

**Figure 4:**
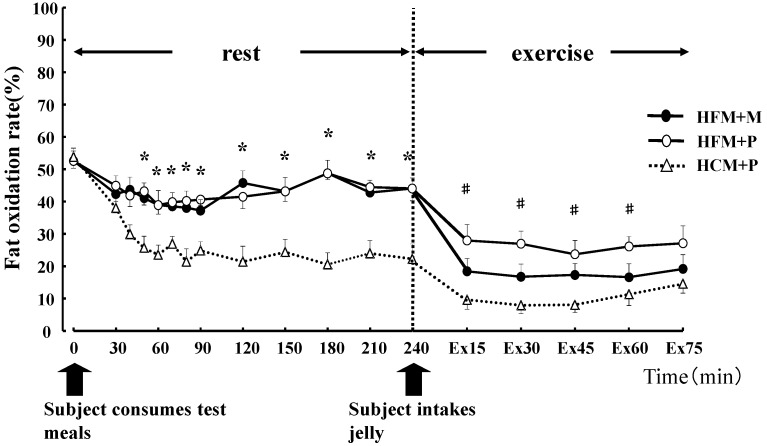
Fat oxidation rates (%) for 240 min at rest and for 75 min during exercise. * indicates when high-fat meal with maltodextrin (HFM+M) and high-fat meal with placebo (HFM+P) values were significantly higher than high-carbohydrate meal and placebo (HCM+P) values (*p* < 0.05). ^#^ indicates when HFM+P was significantly higher than HCM+P (*p* < 0.05). Data are shown asmean ± S.E.

There was no statistical difference in the average values of oxygen consumption at rest (0.28 ± 0.01 L/min, 0.27 ± 0.01 L/min, 0.27 ± 0.01 L/min, in HFM + M, HFM + P, and HCM + P, respectively) or during exercise (2.59 ± 0.16 L/min, 2.59 ± 0.14 L/min, 2.58 ± 0.17 L/min, in HFM + M, HFM + P, and HCM + P, respectively) among the three groups. The RER, carbohydrate and fat oxidization rates are shown in Figure 2, Figure 3 and Figure 4. At rest, subjects in the HFM + M and HFM + P groups showed a lower RER and carbohydrate oxidation rate than subjects in the HCM + P group. During exercise, these values continued to decrease. In contrast, at rest, the fat oxidation rate was higher in the HFM + M and HFM + P groups as compared with subjects in the HCM + P group. During exercise, the fat oxidation rate was higher in the HFM + P group than the HCM + P group.

**Figure 5:**
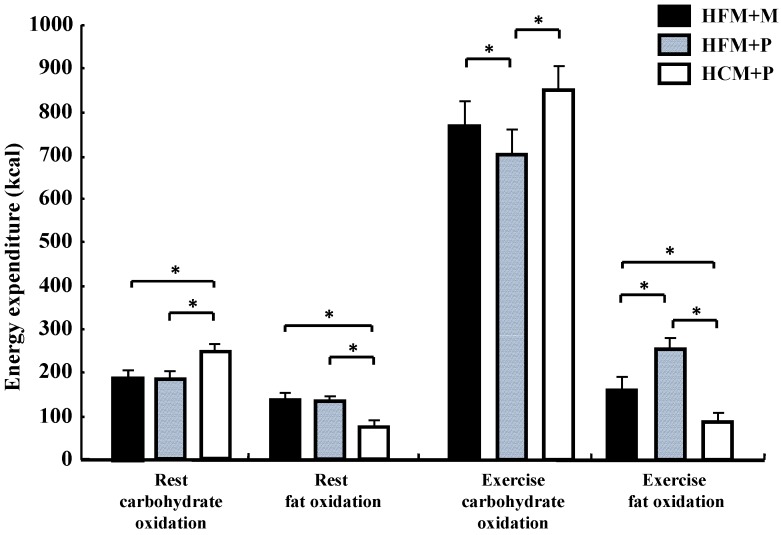
Carbohydrate and fat oxidation for 240 min at rest and for 75 min during exercise following the diets. * indicate significant differences among the trials (*p* < 0.05). (HFM: high-fat meal; HCM: high-carbohydrate meal; M: maltodextrin; and P: placebo jelly).Data are shown as mean ± S.E.

Carbohydrate oxidation (Figure 5) during rest was significantly lower with HFM + M and HFM + P than with HCM + P (*p* < 0.05), while fat oxidation for the same groups (Figure 5) was high (*p* < 0.05). Carbohydrate oxidation (Figure 5) during the 75 min of exercise was significantly lower in subjects in the HFM + P group as compared with those in the HFM + M and HCM + P groups (*p* < 0.05). Fat oxidation (Figure 5) during the 75 min of exercise was significantly higher in groups HFM+P and HFM + M as compared with the HCM + P group (*p* < 0.05). Furthermore, fat oxidation was higher in HFM + P as compared with HFM + M (*p* < 0.05). The total energy expenditure was not substantially different among the three trials.

Carbohydrate oxidation both during rest and exercise was significantly lower (*p* < 0.05) in subjects in the HFM + P group (887 ± 68 kcal) when compared with subjects in the HFM + M (969 ± 62 kcal) and HCM+P (1085 ± 60 kcal) groups. In addition, subjects in the HFM + M group showed a lower carbohydrate oxidation than those in the HCM + P group (*p* < 0.05).

The HR and RPE values during exercise increased as time passed, but no significant differences were found between the three groups.

**Figure 6:**
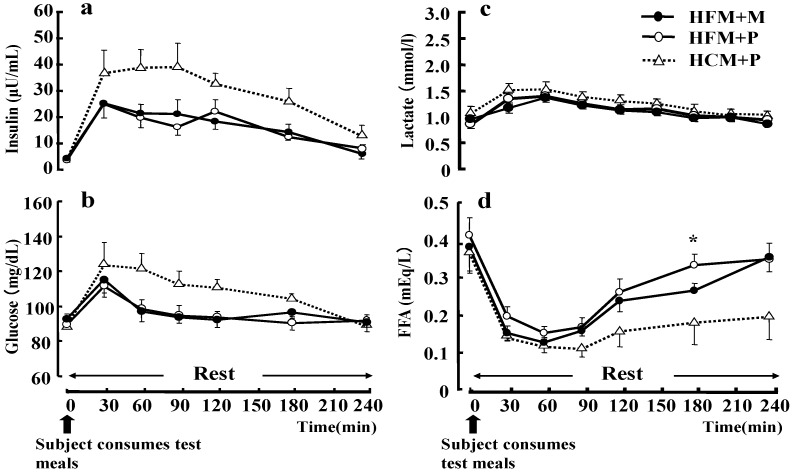
Changes in insulin (**a**), glucose (**b**), lactate (**c**), and free fatty acids (FFA) (**d**) at rest. Data were analyzed using a two-way analysis of variance (ANOVA) with repeated measures. Tukey’s test was used to identify any statistically significant differences. * indicates when high-fat meal with placebo (HFM+P) values were significantly higher than high-carbohydrate meal with placebo (HCM+P) values (*p* < 0.05). (HFM+M: high-fat meal with maltodextrin).Data are shown as mean ± S.E.

No statistical differences were found between the three trials with respect to plasma glucose, serum insulin and LA concentration during rest (Figure 6). However, the serum FFA concentration (Figure 6d) during rest was significantly higher in subjects that had ingested a HFM + P as compared with those who had ingested a HCM + P (*p* < 0.05). 

**Figure 7:**
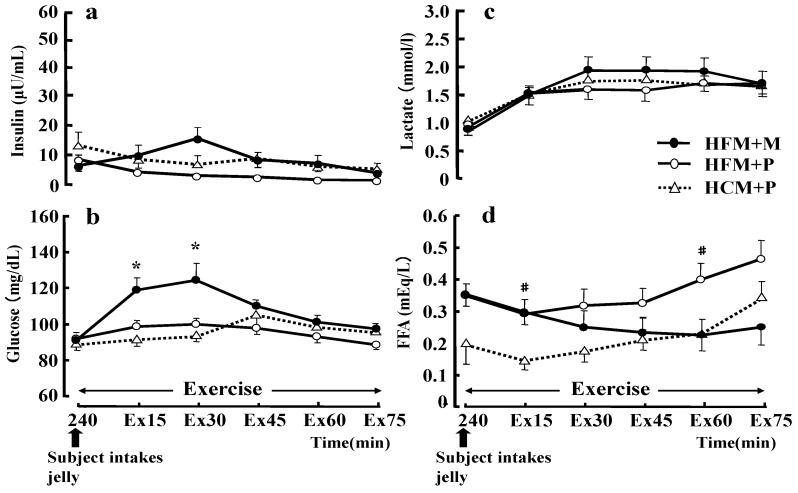
Changes in insulin (**a**), glucose (**b**), lactate (**c**), and free fatty acids (FFA) (**d**) during exercise. Data were analyzed using a two-way analysis of variance (ANOVA) with repeated measures. Tukey’s test was used to identify any statistically significant differences. * indicates when high-fat meal with maltodextrin (HFM+M) values were significantly higher than high-fat meal with placebo (HFM+P) values (*p* < 0.05). ^#^ indicates when HFM+P values were significantly higher than high-carbohydrate meal with placebo (HCM+P) values (*p* < 0.05). Ex15, Ex30, Ex45, Ex60, Ex75 correspond to sample points at 15, 30, 45, 60 and 75 min of exercise, respectively. Data are shown as the mean ± S.E.

The plasma glucose concentration at 15 and 30 min of exercise (Figure 7b) was significantly higher with the HFM + M diet than with the HFM + P diet (*p* < 0.05). However, regarding the serum insulin concentration during exercise (Figure 7a), no differences were found between the three trials. Serum insulin concentrations during exercise were higher for HFM+M (14.9 ± 3.7 μU/mL) than for HFM + P (2.9 ± 0.5 μU/mL) and HCM + P (6.5 ± 3.0 μU/mL) at 30 min of exercise, but two-way ANOVA for repeated measurements did not demonstrate any significant difference. The serum FFA concentrations (Figure 7d) during exercise were significantly higher with HFM + P than with HCM + P at 15 and 60 min of exercise (*p* < 0.05). The LA concentration (Figure 7c) was not significantly different between the three trials during exercise, and the LA concentrations at exhaustion were 2.9 ± 0.3 mmol/L, 2.5 ± 0.2 mmol/L, 3.0 ± 0.2 mmol/L, for HFM + M, HFM + P, and HCM + P groups, respectively.

## 4. Discussion

The main finding of this study was that carbohydrate ingestion subsequent to a meal that is high in fat after 3 days of glycogen loading can enhance the endurance running performance of athletes. Ingestion of a HFM lowers carbohydrate oxidation as compared with a HCM both at rest and during exercise. Although there was no significant difference in seven of the eight subjects, the running times was longer when they ingested a HFM + P as compared with a HCM + P. Moreover, the HFM and carbohydrate ingestion (M) immediately before exercise improved the running time over the other two diets. The blood glucose was markedly increased in subjects in the HFM + M group during exercise; however, the LA was similar for subjects when fed either HFM + P or HCM + P (Figure 7). This result suggests that ingestion of carbohydrate immediately prior to exercise increases glycolysis (Figure 3), and has a very small effect on LA production. 

The intake of HFM before exercise has previously been demonstrated to promote the mobilization of serum FFA and increase lipid metabolism, effectively enhancing fat oxidation during exercise [9]. In this study, HFM + P was associated with increased serum FFA levels and fat oxidation during rest and exercise, when compared with the findings for subjects in the HCM + P group. An increased serum FFA concentration before exercise contributes to the conservation of muscle glycogen during exercise [8,10,11,12]. However, previous studies in humans that investigated the intake of HCM and HFM before exercise [9,13] report no differences in performance during exercise. In this study, despite all subjects believing that HCM is preferable to HFM before exercise, a small improvement in performance was observed in seven out of eight subjects when HFM was ingested.

Thus, the results of this study do not confirm the findings of several previous studies [9,13,25]. However, it is likely that the discrepancy stems from differences in the nutritional status of the subjects prior to exercise, including the level of carbohydrate loading, and differences in the design of the exercise test. Saltin and Hermansen [5] and Sherman *et al.* [6] demonstrated that in order to maximize the amount of muscle glycogen stored, it is essential to ingest a HCM, with a carbohydrate content of 30 kcal per body mass, for 2 to 3 days before exercise. Therefore, for carbohydrate loading before a race, our subjects were served a meal containing 71% carbohydrate (carbohydrate content of 32.5 kcal per body mass) at each meal for 3 days. However, in previous studies [9,13], the subject ingested meals for 2 to 3 days before the tests included only 20.7 to 25.6 kcal per body mass of carbohydrate. The muscle glycogen storage might have more available in those studies. Second, an appropriate exercise test should be used when evaluating pre-exercise feedings. High- to moderate-intensity exercises, such as time trials, were generally used in previous studies [25]. In our study, the subjects performed an exhaustion test, after a long duration run in order to induce lower glycogen level. The LA concentration was about 3 mmol/L (Figure 7) at exhaustion; therefore, it is unlikely that LA buildup limits the running in our subjects. This is the original point of our study. Consequently, we suggest that the exercise regime employed in this study better evaluates the effect of diet on endurance performance such as marathons. Furthermore, this study excluded the inter-individual differences in each trial by the random order crossover method, and subjects ingested the carbohydrate or placebo jelly in a blind fashion. The study design thus improves the confidence of these results.

The exercise time was extended most when carbohydrates were loaded immediately before exercise after HFM was ingested. In previous studies [14,15,16], when glucose was supplied during exercise, the blood glucose concentration was maintained and performance improved, as compared with subjects that did not receive glucose. Maintaining the blood glucose concentration by supplying carbohydrates during exercise can generate large amounts of energy from glucose and prevent the early exhaustion of muscle glycogen [14,18]. In this study, for practical reasons, the administration of large amounts of carbohydrates was attempted 3 min before exercise and not during exercise. The administration of carbohydrates may promote excessive insulin secretion and excessive muscle glycogen oxidation, as seen in Costill *et al.* [8]. Further, the intake of carbohydrates (75 g) 45 min before starting exercise significantly increased insulin concentrations during exercise and increased the use of glycogen. However, we believe that if carbohydrates are ingested immediately before exercise, the excessive secretion of insulin can be countered and suppressed by the insulin secretion inhibitory effects upon starting exercise [26]. In fact, in the HFM + M trial in this study, although large amounts of carbohydrates (about 102 g) were ingested 3 min before starting exercise, and blood-glucose levels at 15 and 30 min of exercise levels were significantly increased as compared with subjects in the HFM + P group (Figure 7b), there were no significant changes in the insulin level during exercise (Figure 7a). During 30 min of rest after the ingestion of a HFM (containing 75 g (30%) carbohydrate), the insulin concentration values increased up to 25.2 μU/mL (Figure 6a). However, the intake of 102 g of carbohydrates as jelly immediately before exercise increased the insulin concentration value only to 14.9 μU/mL after 30 min of exercise (Figure 7a). As a result, the serum FFA concentration (Figure 7d) was maintained at high levels, while the blood glucose concentration continued to increase during the initial stages of exercise; this may therefore contribute to the high fat oxidation during exercise in the HFM groups as compared with the HCM group. The initial increase of blood glucose in the HFM + M group contributes to the oxidation of carbohydrate sources other than muscle glycogen during exercise. Therefore, carbohydrates taken immediately before exercise improved the endurance performance by replacing carbohydrates that had been consumed during rest before exercise in the liver and blood, while also storing exogenous energy other than muscle glycogen in the body when starting exercise.

## 5. Conclusions

The present study indicates that following 3 days of glycogen loading, a HFM and subsequent ingestion of a small portion of carbohydrate jelly prior to exercise enhances the performance of athlete endurance running. This diet pattern is favorable for physical conditioning of athletes preparing for and competing in a marathon race.
